# Physical, Mechanical, and Morphological Properties of Treated Sugar Palm/Glass Reinforced Poly(Lactic Acid) Hybrid Composites

**DOI:** 10.3390/polym13213620

**Published:** 2021-10-20

**Authors:** S. F. K. Sherwani, E. S. Zainudin, S. M. Sapuan, Z. Leman, A. Khalina

**Affiliations:** 1 Advanced Engineering Materials and Composites Research Centre (AEMC), Department of Mechanical and Manufacturing Engineering, Universiti Putra Malaysia (UPM), Serdang 43400, Malaysia; faisalsherwani786@gmail.com (S.F.K.S.); zleman@upm.edu.my (Z.L.); 2 Laboratory of Biocomposite Technology, Institute of Tropical Forestry and Forest Products (INTROP), Universiti Putra Malaysia (UPM), Serdang 43400, Malaysia; khalina@upm.edu.my

**Keywords:** mechanical properties, sugar palm fiber, poly(lactic acid), alkaline treatment, benzoyl chloride treatment, hybrid composites

## Abstract

This research was performed to evaluate the physical, mechanical, and morphological properties of treated sugar palm fiber (SPF)/glass fiber (GF) reinforced poly(lactic acid) (PLA) hybrid composites. Morphological investigations of tensile and flexural fractured samples of composites were conducted with the help of scanning electron microscopy (SEM). Alkaline and benzoyl chloride (BC) treatments of SPFs were performed. A constant weight fraction of 30% total fiber loading and 70% poly(lactic acid) were considered. The composites were initially prepared by a Brabender Plastograph, followed by a hot-pressing machine. The results reported that the best tensile and flexural strengths of 26.3 MPa and 27.3 MPa were recorded after alkaline treatment of SPF, while the highest values of tensile and flexural moduli of 607 MPa and 1847 MPa were recorded after BC treatment of SPF for SPF/GF/PLA hybrid composites. The novel SPF/GF/PLA hybrid composites could be suitable for fabricating automotive components.

## 1. Introduction

Due to environmentally friendly customers’ desires to save the earth, recently there has been a growing interest in using renewable resources and biodegradable products. For the development of polymer composites, many major industries have focused on using natural fibers. This is owing to the benefits offered by natural fibers (e.g., they are cost-effective, dense, easy to obtain, environmentally safe, non-toxic, durable, reusable, biodegradable, abrasion-resistant, have high strength and modulus, and are simple to process) [1,2]. Natural fibers’ biodegradability makes them ideal for reinforcement in polymer composites [3,4]. Both natural and synthetic fibers can be used to create hybrid composites. This combination demonstrates outstanding structural and mechanical properties [5,6]. Recently, sugar palm has been considered by many studies as a desirable natural fiber due to its easy cellulose separation from other components [7]. Sugar palm (*Arenga pinnata Wurmb. Merr*) is found abundantly in Malaysia, Indonesia, India, and Thailand [8]. SPF has the advantages of having low density, biodegradable, lack of toxicity, low cost, non-abrasive nature, and long-lasting [9,10].

In the world of biodegradability, efforts in commercializing PLA polymers have been taking place since the last decade. PLA is a thermoplastic bio-based product obtained from fermentation processes of natural agricultural raw materials (starch and sugar) [11,12]. PLA is recyclable, readily decomposable, has cheap manufacturing costs, and is commercially available [13]. These features enable PLA to replace petroleum-based polymers for several biomedical, textile, plastic, 3D printing materials, and packaging applications [14,15]. However, some drawbacks of PLA include its high price and water sensitivity, low crystallinity rate, and fragility [16]. Many researchers aim towards overcoming these limitations, commonly by mixing PLA with natural fibers [17].

Moisture absorption is a major problem with natural fibers that negatively affects the strong interfacial bonding between a fiber and a matrix. This problem can be solved by fiber pre-treatment, which eliminates lignin and other related materials, improving the interfacial bonding between the fiber and the matrix [18,19]. Treating the lignocellulosic fiber for improved adhesiveness and good stress transfer from the matrix to the rigid fiber will increase the performance of different hybrid composites. Since hydrophilic fiber does not tightly bond with a hydrophobic polymer, weak fiber-matrix bonding degrades composite strength [8,18]. Treatment of the fiber is needed to enhance bonding between the fiber and the matrix. After the treatment, the fiber has reduced moisture absorption, increased bonding, and, most significantly, both enhanced physical and mechanical properties. Alkaline treatment is one of the most effective chemical treatments for fiber surface modification [18,19,20]. An alkaline treatment procedure has proven to be a successful treatment for extracting waxy substances and impurities [20,21]. As a result, the fiber’s surface becomes rough, allowing for greater adhesion with the polymer. This treatment affects the fiber in two ways. Firstly, hydrogen bonding in the network structure is disrupted, enhancing the surface roughness. Secondly, lignin, wax, and oils are eliminated from the surface, enhancing the exposure of cellulose to the fiber surface. This results in increasing the number of possible reaction sites [22]. However, alkaline treatments have the added problem of fiber degradation at high concentrations, which may be addressed with a moderate chemical treatment such as benzoyl chloride.

The fiber treatment with BC decreases the hydrophilic nature of natural fibers and strengthens fiber attachment to the matrix, improving the bio-composites’ strength [23,24]. Alkali pre-treatment is used during the benzoylation process. When further treated with BC, these alkaline pretreated fibers lead to the —OH groups of the cellulose fibers overtaken by benzoyl groups and render them hydrophobic [22]. Extractable materials such as waxes, lignin, and oil-coating materials are removed at this stage, exposing more volatile hydroxyl groups on the fiber surface. The fiber’s —OH groups are substituted by the benzoyl groups, which binds to the cellulose backbone [25]. Prabhu et al. [24] addressed that the benzoylation of fiber improved adhesion to the fiber-matrix, significantly improved composite strength and decreased water absorption. The treatment improved the wettability of *Impomea pes-caprae* fiber and epoxy composites, resulting in stronger bonding and increased overall composite strength [26]. Palmyra palm-leaf stalk fiber composites were also treated with BC, which improved tensile strength and modulus by 60% [27]. The BC treatment strengthened fiber and matrix adhesion, improved strength, and reduced the water absorption character of the whole composite by reducing SPF’s hydrophilic nature and strengthening the blend with epoxy resin matrix [25,28]. Currently, Mohd Izwan et al. [29] investigated SPF benzoylation treatment and found that the highest tensile strength was achieved after 15 min of soaking time, indicating good SPF properties for use as reinforcement in composites.

Several studies were conducted on the hybridization of natural–natural fibers, natural–synthetic fibers and synthetic–synthetic fibers in a single matrix, showing that hybrid composites have been promising to enhance mechanical properties [30,31,32,33]. However, in hybrid composites, fiber loading is typically limited to a maximum of 50% [34]. Atiqah et al. [35] studied the mechanical properties of kenaf-glass reinforced unsaturated polyester hybrid composites. They found that combining these fibers improved the tensile, flexural, and impact properties. Afzaluddin et al. [36] developed SPF/GF reinforced TPU hybrid composites and studied their physical, tensile, flexural, and impact properties. They reported that tensile and impact properties of the hybrid composites were increased with the increase of the content of SPF (%) compared to GF reinforced composites. Still, the flexural properties were improved when the content of GF was increased. Atiqah et al. [37] studied the effects of silane and alkaline treatment of fiber on the tensile, flexural, and impact properties of SPF/GF/TPU hybrid composites and found that treated SPF can increase the mechanical properties of hybrid composites. In another study, Atiqah et al. [38] reported that physical properties could be improved by alkaline treatment of SPF in fabricating the SPF/GF/TPU hybrid composites. Recently, Radzi et al. [20] determined the physical and mechanical properties of roselle/SPF reinforced thermoplastic polyurethane hybrid composites and revealed that the alkaline treatment on RF/SPF hybrid composite has improved the mechanical properties of hybrid composite and proven the RF/SPF composite suitability for making automotive parts.

To the best of our knowledge, no study on treated SPF/GF/PLA hybrid composites has been reported in the literature. Novel combinations of treated SPF/GF are selected as effective reinforcements PLA hybrid composite due to their positive effects on improving physical, mechanical, and morphological properties. Due to the presence of SPF and PLA bio-degradable plastic manufactured from plant-based resources such as corn starch or sugarcane, this hybrid composite is environmentally friendly. The hybrid composites had a constant SPF/GFs weight fraction of 30%. Sherwani et al. [39] demonstrated that a 70:30 (PLA/SPF) ratio exhibits excellent mechanical properties, especially tensile and flexural properties. Hence, a 70% PLA to 30% fiber ratio is considered in this research. The effect of alkaline and benzoyl chloride treatment of SPFs on PLA were also determined by Sherwani et al. [40] and reported that the optimum percentage is 6% alkaline and the best soak duration is 15 min for enhancing the mechanical properties of SPF/PLA composites. As a result, a 6% alkaline and 15-min benzoyl chloride treatment is considered here. The aim of this paper is to evaluate the physical, tensile, flexural, and impact properties as well as the post-tensile and flexural morphological tests, aiming to propose the best natural/green hybrid composite formulation for engineering material which can be used for the applications of automotive (especially motorcycle) components manufacturing. With the help of this research, an environmentally friendly biodegradable material may be developed for possible application in the manufacture of motorcycle components.

## 2. Materials and Methods

### 2.1. Materials

Sugar palm fiber (length of fiber up to 1.19 m, average diameter 0.5 mm, the density of raw SPF 1.2–1.3 gm/cm^3^, and tensile strength 15.5 MPa [41]) were purchased from Kampung Kuala Jempol, Negeri Sembilan, Malaysia. The poly(lactic acid) (NatureWork 2003D) (density 1.25 gm/cm^3^ at 21.5 °C, yield tensile strength of 52 MPa, and melting point of 170 °C), BC with reagent plus 99%, ethanol, and E-glass fiber (properties of E-glass is shown in Table 1) were delivered by Mecha Solve Engineering, Petaling Jaya, Selangor, Malaysia. Sodium hydroxide (NaOH) pellet was delivered by Evergreen Engineering and Services, Taman Semenyih Sentral, Selangor, Malaysia.

The electrical resistivity of E-glasses at room temperature is exceptionally high. E-glass compositions by weight percentage are SiO_2_-60; Al_2_O_3_-9; MgO-4; CaO-27.

### 2.2. Preparation of Sugar Palm Fiber

A crusher machine was used for crushing a bundle of SPF. The dry SPF was graded to a length of 10 mm to 15 mm using a crusher. The fiber was then cleaned several times with water to remove impurities. The SPF was left outdoor for 24 h before being dried in an air-circulating oven at 60 °C.

### 2.3. Chemical Treatments

#### 2.3.1. Alkaline Treatment

Alkaline treatment of natural fibers was used to eliminate surface impurities as well as hemicelluloses within the fibers [20]. In our study, 50 g of SPFs was soaked in a 6% *w/v* alkaline solution of 1000 mL for 1 h at 25 °C. After that, they were immersed with an acetic acid solution till a neutral pH value was obtained prior to washing with distilled water and oven-drying for 24 h at 60 °C. The dried SPF was placed into zipper plastic storage bags. Table 2 shows the chemical composition of SPF and alkaline treated SPF.

#### 2.3.2. Benzoyl Chloride Treatment

**Scheme 1:**
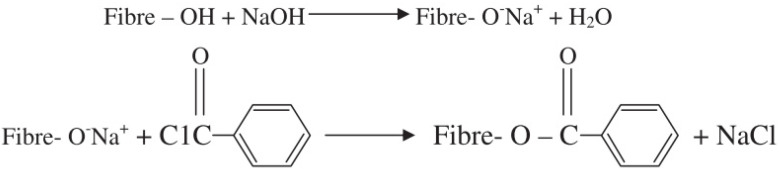
The reaction between the cellulosic —OH groups of fiber and BC. Alkaline pretreated fibers, when further treated with BC, the OH groups of the cellulose fibers are replaced by benzoyl groups which reduced the hydrophilic character of fiber.

50 g SPF was immersed in 18% NaOH solution for 30 min, then washed SPF twice with tap water. The SPF was suspended in a 10% NaOH solution and vigorously agitated for 15 min in 50 mL BC. Once again, SPF was washed in water, filtered, and dried at 25 °C. SPFs is normally immersed in ethanol for an hour before being washed, filtered, and dried in a 60 °C oven for 24 h [28,29]. The reaction between the cellulosic —OH groups of fiber and BC is given in Scheme 1.

### 2.4. Fabrication of SPF/GF Reinforced PLA Hybrid Composites

**Figure 1:**
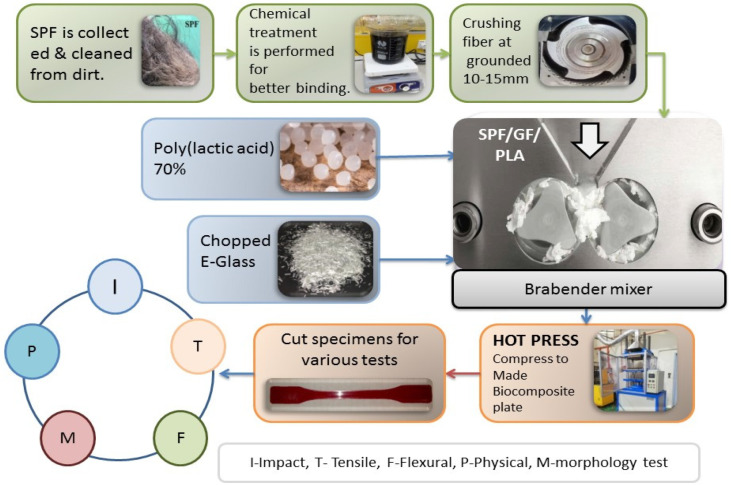
Detailed description of the methodology flow diagram.

The melting compounding and hot press moulding methods were used to prepare the SPF/GF reinforced PLA hybrid composites. The 10–15 mm sugar palm fiber, 12.5 mm chopped E glass fiber, and PLA pellets were dried at a temperature of 60 °C in electric ovens for 48 h. Nine sets of SPF/GF composites (30/0, 20/10, 15/15, 10/20, and 0/30) wt % reinforced PLA were developed as seen in Table 3. Based on past research [34,36,44], such a ratio was considered. In a Brabender Plastograph (co-rotating twin-screw extruder), untreated/treated SPF and chopped E-glass fibers reinforced PLA were mixed for 10 min at 160 °C with a rate of 50 rpm to ensure consistent mixing. These samples were then crushed using a crushing unit. After Brabender mixing, to reduce voids or gaps, the composite samples must be placed in an electric oven for 24 h at 60 °C before the hot press. A compression moulding Techno Vation machine model 40 tons was used for hot-press moulding. These samples were pre-heated for 7 min at 170 °C before being completely pressed for 6 min. There were three vent cycles to remove voids in the composites. During the final cycle, the cold-pressed time was 6 min at 25 °C. Figure 1 describes the detailed methodology of this research.

## 3. Characteristics of SPF/PLA/GF Hybrid Composites

### 3.1. Density

Non-hybrid and hybrid composites’ experimental densities were measured using the Mettler Toledo XS205 electronic densitometer as per the ASTM D792 standard [45]. Equation (1) was used to evaluate the theoretical density of the composite,

(1) $$\mathrm{Theoretical}\;\mathrm{density}\;\rho_{ct}=\frac{1}{\left(\frac{w_{sf}}{\rho_{sf}}\right)+\left(\frac{w_{gf}}{\rho_{gf}}\right)+\left(\frac{w_{m}}{\rho_{m}}\right)}$$

where $w_{sf},\;w_{gf}$, and $w_{m}$ represent the weight fraction of the SPFs, glass fibers, and matrix, respectively, while $\rho_{sf}\;,\;\rho_{gf}\;,\mathrm{and}\;\rho_{m}$ denote the density of the SPF, glass fibers, and matrix, respectively. Subsequently, the volume fraction of voids in the percentage of the composites is calculated by using Equation (2) [18].

(2) $$\mathrm{Volume}\;\mathrm{fraction}\;\mathrm{of}\;\mathrm{void}\;V_{void\;}=\frac{\rho_{ct}-\rho_{comp.\;\;exp.}}{\rho_{ct\;}}\;\times 100$$

$\rho_{comp.\;exp.}$—Composite experimental density.

### 3.2. Moisture Content

All of the non-hybrid and hybrid composites were tested for moisture content analysis. In an oven, the composites were heated to 100 °C for 24 h. To calculate the moisture content, the weights of the composites were determined before (*M_bh_*, gram) and after (*M_ah_*, gram) being placed into the oven. The following Equation (3) was used:

(3) $$\mathrm{Moisture}\;\mathrm{content}\;(\%)=\frac{M_{bh}-M_{ah}}{M_{bh}}\;\times \;100$$

### 3.3. Water Absorption Test

Non-hybrid and hybrid composites were tested for water absorption (*WA*) using the ASTM D570 standard [46]. From the composite plate, a rectangular geometry sample measuring 10 mm × 10 mm × 3 mm was removed. The weight of the composite was calculated as an initial mean before it was immersed in water, *W_iw_*, and *W_fw_* as a final stage mean after a day of immersion in water at about 25 °C. The test was conducted for eight days. The following Equation (4) was used for calculating water absorption:

(4) $$\mathrm{Water}\;\mathrm{Absorption}\;(\%)=\frac{\left(W_{fw}-W_{iw}\right)\;}{W_{iw}}{\;}_{\;}\times \;100$$

### 3.4. Thickness Swelling

The thickness swelling (*TS*) of all non-hybrid and hybrid composites with measurements of 10 mm × 10 mm × 3 mm was evaluated. Using a digital vernier caliper, the thickness was calculated as *T*_1*b*_ (before) and *T*_2*a*_ (after) they were immersed in water [20]. The *TS* reading was taken for eight days and was determined using Equation (5).

(5) $$\mathrm{Thickness}\;\mathrm{Swelling}\;(\%)=\frac{T_{2a}-T_{1b}}{T_{1b}}\;\times \;100$$

### 3.5. Tensile Test

An Instron3366 universal testing machine (UTM, University Ave Norwood, Norwood, MA, USA) was used to conduct a tensile test in accordance with ASTM Standard D638-10 [47]. The gauge length of the non-hybrid and hybrid composites was 80 mm, and the crosshead velocity was 2 mm/min, using a 5 KN load cell. Five samples measuring 150 mm × 25 mm × 3 mm were tested. The average of the five samples provided the final result.

### 3.6. Flexural Test

Using an Instron 3365 dual column tabletop UTM with a span length of 50 mm and a crosshead speed of 12 mm/min, the flexural properties of non-hybrid and hybrid composites were tested according to the ASTM D790 standard [48]. Five composite samples measuring 127 mm × 12.7 mm × 3 mm were obtained from the composite plate. The average of the five samples provided the final result.

### 3.7. Impact Test

ASTM D256 (2010) [49] Standard Izod impact test samples measuring 65 mm × 15 mm × 3 mm were taken out of the non-hybrid and hybrid composite plates. Rayran RR/IMT/178 Izod impact testers were used for the impact study. For each sample, the average of the three readings was calculated for the final result of the impact test. Five identical samples were rigidly placed in a vertical position with each type of composite and struck in the center of the instruments with a pendulum with a force of 10 J. The impact had 2.75 J energy and a velocity of 3.46 m/s.

### 3.8. Morphological Investigations

SEM (Coxem-EM-30AX+) was used to examine broken surfaces of tensile and flexural samples for morphological analysis. Scanning electron microscopy (SEM) with a working distance of 14.7 mm, a 58 A emission current, and a 20.0 kV acceleration voltage was used. The samples were coated with a fine gold film for electrical conductivity, improving the image resolution appreciably.

### 3.9. Fourier Transform Infrared (FTIR)

The FTIR spectra were used to examine the presence of functional groups existing in non-hybrid and hybrid composites. The samples were segregated into 10 mm × 10 mm × 3 mm squares and then analyzed with a power FTIR spectrometer with attenuated total reflectance (ATR) (Nexus Analytics-Isio 713601, Petaling Jaya, Selangor Darul Ehsan, Malaysia). The wavenumber spectrum for the spectra was 4000 cm^−1^ to 500 cm^−1^, and the measurements were taken with a resolution of 4.0 cm^−1^ and 16 scans per sample.

### 3.10. Statistical Analysis

SPSS software was used to perform an analysis of variance (ANOVA) on the obtained experimental results. ‘Duncan’s test was employed to conduct a mean comparison at a 0.05 level of significance (*p* ≤ 0.05).

## 4. Results and Discussion

### 4.1. Density

**Figure 2:**
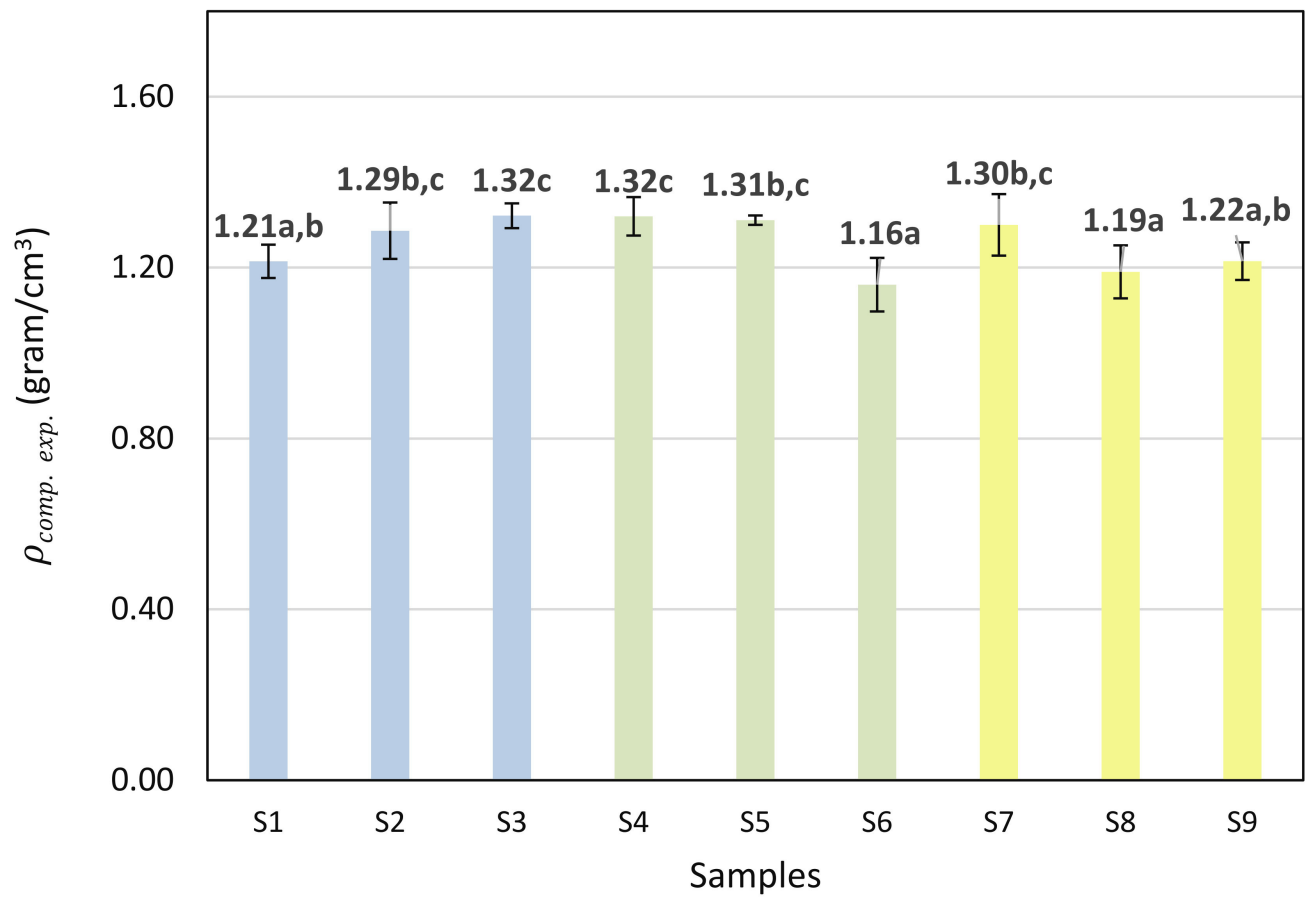
Composite experimental density $\rho_{comp.\;exp.}$ vs. samples for non-hybrid and hybrid SPF/GF/PLA composites. * Values with different letters in the figures are significantly different (*p* < 0.05).

Figure 2 illustrates the density ($\rho_{comp.\;exp.}$) value measurement for non-hybrid and hybrid SPF/GF/PLA composites. The density result showed that the density was improved by adding GF to the SP/PLA composites from 1.21 to 1.32 gm/cm^3^. Comparing S1, –S3 composites, the density of S3 exhibited the highest value of 1.32 gm/cm^3^, concluding that the hybridization of SPF/PLA composite increases the density of the composite. A similar finding was also revealed from a previous work done by Afzaluddin et al. [36] that density increment resulted from the addition of glass fiber into SPF/GF/TPU hybrid composites. This was due to the fact that GF has a higher density than SPF. The densities of untreated S3 and treated S5, S8 SP/GF hybrid composites were 1.32, 1.31, and 1.19 gm/cm^3^. The density slightly decreased after treatment of SPF. In previous studies by Atiqah et al. [38] and Merlini et al. [50], density reductions of treated fibers were also recorded. Comparing alkaline treated S4–S6, and BC treated S7–S9 hybrid composites, density results showed that the density decreased slightly as the SPF content was increased, irrespective of whether the SPF has been alkaline or BC treated. As SPF wt % increased, the hydrophilic nature of the SPF made it more difficult to develop the composite properly. This led to voids in the hybrid composites, decreasing the composite density. A similar finding was supported by Safri et al. [51], where the density was measured for SPF/GF/epoxy hybrid composites.

### 4.2. Moisture and Void Contents

Table 4 shows moisture contents, theoretical/experimental composites density, and void contents of non-hybrid and hybrid SPF/GF/PLA composites. In general, the experimental and theoretical densities differed from each other due to a significant influence of voids and pores in the composite towards the behavior of the composite [40]. It is clear from Table 4 that, with the help of glass hybridization, the percentage of voids decreased, as confirmed by SEM images in the morphological investigation. The finding was also supported by the results from work by Radzi et al. [20]. The S4–S6 (alkaline treated) hybrid composites exhibited lower %voids than S7–S9 (benzoyl treated) hybrid composites. This was due to the good compatibility of SPF and GF with the PLA matrix. According to Jawaid et al. [36], voids formation is due to the incompatibility of natural fiber with matrix to displace all the trapped air which is entrained during fabrication of hybrid composite and incomplete wetting out of the fibers by the matrix. This research also reported that after Brabender mixing, the composite samples need to be placed in an electric oven for 24 h at 60 °C before the hot press. Otherwise, more voids are visible due to their moist content.

### 4.3. Water Absorption Analysis

**Figure 3:**
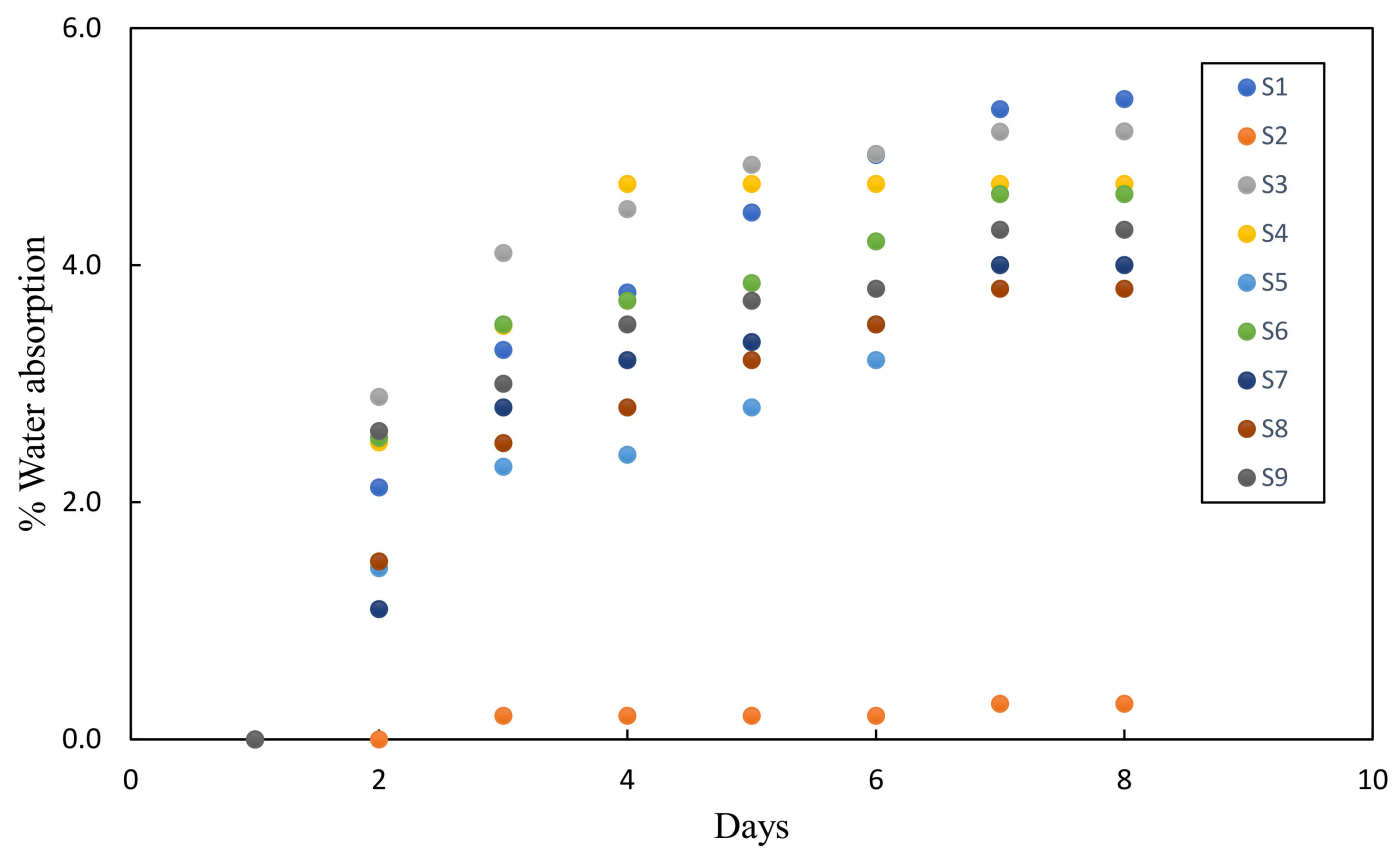
% Water absorption (*WA*) versus samples for non-hybrid and hybrid SPF/GF/PLA composites.

Figure 3 shows the effect of treatments on the values of water absorption (*WA*) of non-hybrid and hybrid SP/GF/PLA composites. The higher SPF content (S1 composite) resulted in a higher *WA* value. The S2 (GF/PLA) composite was the lowest *WA* compared to other non-hybrid or hybrid composites. Among the SPF/GF/PLA hybrid composites, S5 and S8 composites had the lowest *WA* values as both composites exhibited the same 15 wt % SPF. S5 composite was alkaline treated, indicating that alkali hydroxyl (—OH) groups inside the molecules were broken down and when reacted with H_2_O molecules and left the fiber structure. The other reactive molecules produce fiber-cell-O-Na groups in the molecular cellulose chains. This reduced hydrophilic hydroxyl groups and increased the water absorption resistance of the fiber. A considerable number of hemicelluloses, lignin, pectin, wax, and oil were also extracted after alkaline treatment [25], while BC treated S8 composite indicated that the fibers were treated with BC after pre-alkaline treatment. The —OH fiber groups were substituted by the benzoyl group and bonded to the backbone of the cellulose. This resulted in the increased hydrophobicity of the fiber and enhanced matrix adhesion. The following is the order of the decreasing value of hybrid and non-hybrid SPF/GF/PLA composites’ water absorption S2< S8< S5< S7< S9< S6< S4< S3< S1. After the 6th day, no change was observed in *WA*. For hybrid composite, the higher loading of natural fibers was considered to have more *WA*, whereas the higher GF loading was related to lower *WA*. This was also consistent with the past work of Afzaluddin et al. [36], who found that water resistance increased with the addition of GF in the SPF/TPU composites.

### 4.4. Thickness Swelling

**Figure 4:**
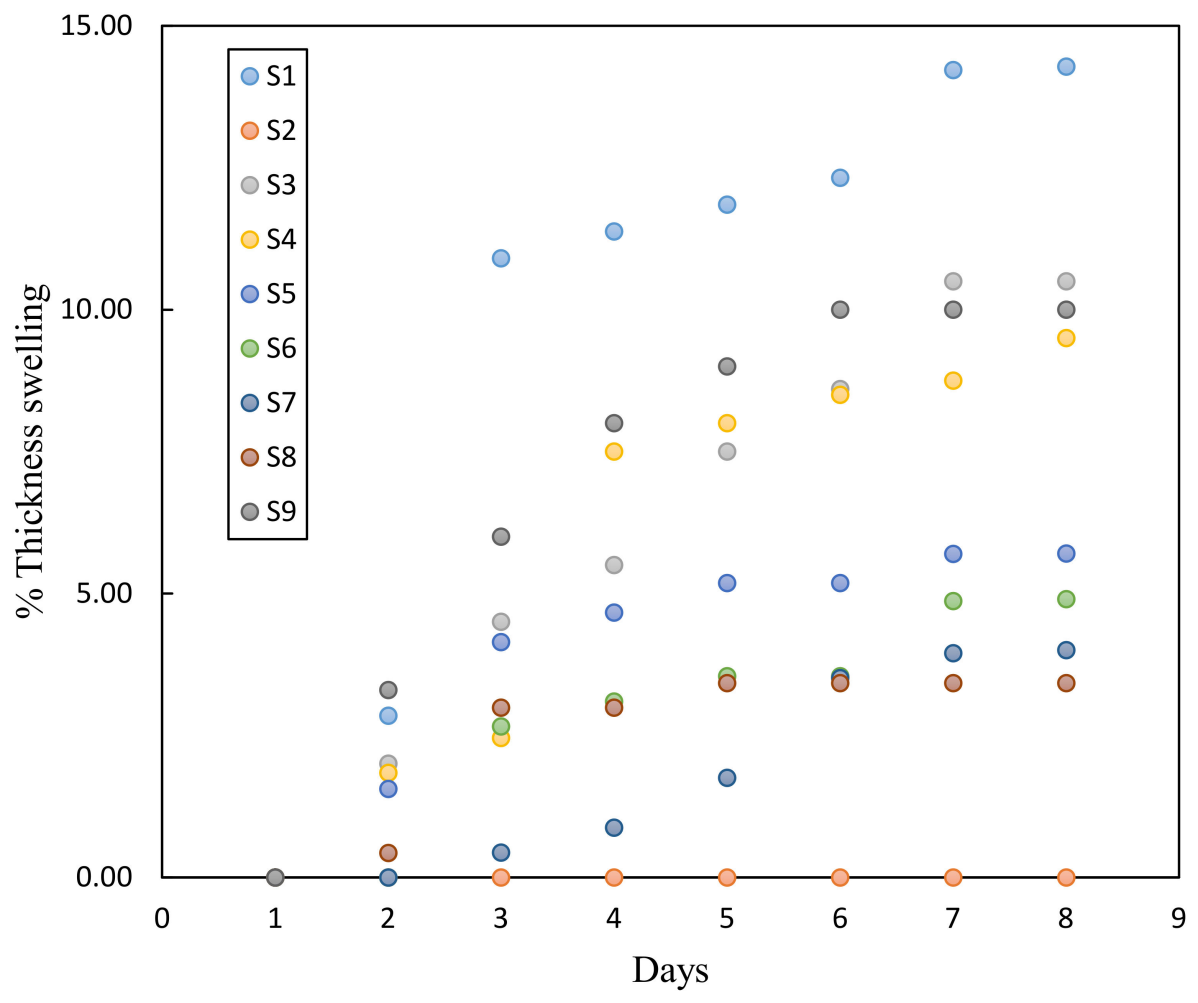
% Thickness swelling (*TS*) versus samples for non-hybrid and hybrid SPF/GF/PLA composites.

Figure 4 presents the effect of treatments on the values of thickness swelling (*TS*) of non-hybrid and hybrid SP/GF/PLA composites. With the increase in *WA*, the thickness swellings of all the composites were increased. The higher the percentage of GF, the lower the *TS* value, while the higher the SPF content, the higher the *TS* value. This was due to the fact that GF possessed water resistance ability while SPF has hydrophilic nature [52]. The highest *TS* value was shown by untreated S1 (SPF/PLA) composite, having maximum content of SPF. In the S2 composite, no *TS* value was observed since it contained only GF and PLA. Among the hybrid composite, the maximum *TS* value was observed in the S3 (untreated SPF/GF/PLA) hybrid composite. The thickness swelling was due to the prolonged immersion duration, where more water molecules were bonded to the hydrogen bonds of fiber. This *TS* value was randomly decreased after alkaline treatment due to the reduction in the micropores and collapsing capillaries, as well as the removal of wax and impurities at the fiber surface after the treatment. This predicted reduced water retention, as indicated by lessening the amount of water absorbed by the fiber. This reduced the *TS* value of the hybrid composite. Due to this cause, as the alkaline treated SPF content was increased, the *TS* value decreased. A similar finding was reported by N.B.M. Hafidz et al. [53] after alkaline treatment of palm oil fiber composites as well as kenaf fiber composites.

The reduction of *TS* was also observed after BC treatment of SPF for hybrid composite. S8 composite showed the lowest *TS* value among all hybrid composite. For the S9 composite, as the benzoyl treated SPF content was increased to 20 wt %, the *TS* value increased due to the disruption of lignin and polysaccharides during treatment that enhanced cellulose concentration. The cellulose chemical structure is composed of hydroxyl that is accessible to water. This directly stimulated the *WA*, thus increased the *TS* [51].

### 4.5. Tensile Testing

**Figure 5:**
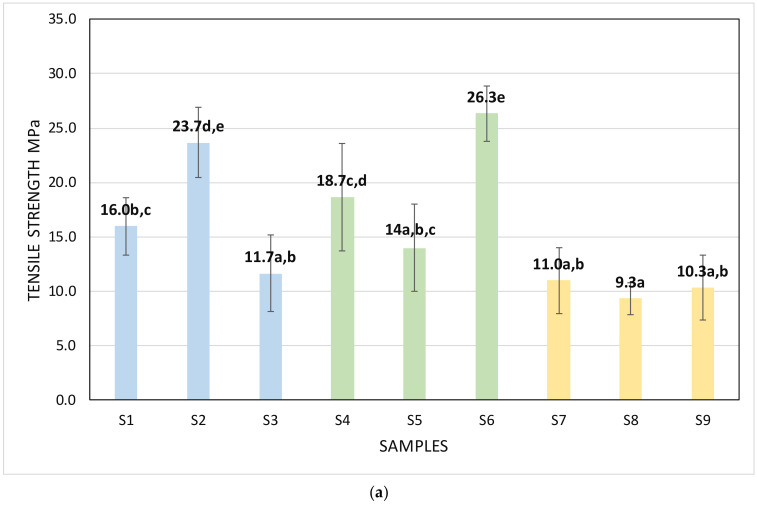

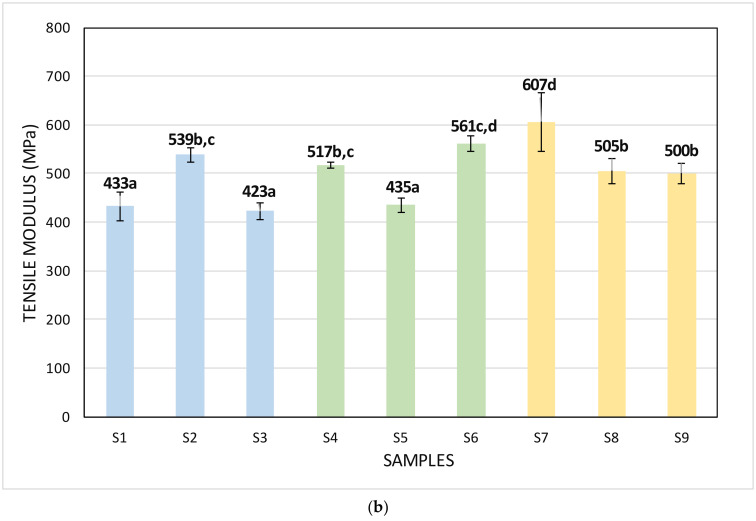
(**a**) Tensile strength versus samples of non-hybrid and hybrid SPF/GF/PLA composite. (**b**) Tensile modulus versus samples of non-hybrid and hybrid SPF/GF/PLA composite. * Values with different letters in the figures are significantly different (*p* < 0.05).

Figure 5a,b shows the effect of treatment on tensile strength and modulus of non-hybrid and hybrid SPF/GF/PLA composites. Hybrid composites were used for various weight percentages of SPFs and GFs. Hybrid composites had a combined weight percentage concentration of fibers (SPF/GF) fixed at 30%, whereas PLA was at 70%. Figure 5a confirmed that by incorporating the GFs, the tensile strength of the PLA composite was increased significantly. From Table 1, the tensile strength of GF was more than SPF, while the tensile strengths of S1 (SPF only) and S2 (GF only) reinforced PLA composite were 16.0 MPa and 23.7 MPa, which indicated that the tensile strength on the addition of GFs was better than untreated SPF. It was observed that the addition of alkali-treated SPF increased the tensile strength of the hybrid SPF/GF reinforced PLA composites. The highest tensile strength of 26.3 MPa was shown by the S6 composite among all hybrid composites. According to Afzaluddin et al. [36], the tensile strength of SPF/GF reinforced TPU hybrid composites could be increased by adding SPF. Among S4–S6 (alkaline treated) hybrid composites, S6 exhibited a maximum tensile strength of 26.3 MPa, while S5 and S4 demonstrated only 14 MPa and 18.7 MPa, respectively. This proves that the interfacial bonding between SPF and PLA matrix had improved after alkaline treatment. S6 hybrid composite exhibited maximum tensile strength that might be due to GF (10% loaded); therefore, SPF can effectively transfer the load from the GF on this particular composition [30]. When alkaline treated SPF/GF/PLA hybrid composites were compared with benzoyl treated SPF/GF/PLA hybrid composites, it is clearly shown in Figure 5a that the alkaline treatment was the best treatment approach used for the improvement of tensile strength. This was due to the improvement in the interface bonding by giving rise to additional sites of mechanical interlocking, facilitating the interpretation of fiber-matrix at the interface. The alkaline treatment of fiber increased the binding properties of the surface by eliminating natural and artificial impurities, which created rough surface topography [30]. This finding was also supported by the morphological investigation of this research.

S3, S5, and S8 had the same composition of 15/15/70 wt % SPF/GF/PLA. The only difference they possessed was the untreated or treated SPF. The analysis showed that the tensile strength of S5 was improved by 27% compared to the S3 hybrid composite. The tensile strength of the alkaline treated SPF/GF/PLA was higher than untreated hybrid composites due to the strong interfacial bonding between treated fiber with PLA matrix. A similar study was carried out by Atiqah et al. [3], that the surface treatment of fiber improved the tensile properties of SP/GF reinforced TPU hybrid composites. The minimum value of tensile strength was 9.3 MPa, which was recorded for S8. The inclusion of BC treated SPF in SPF/GF/PLA hybrid composites caused a remarkable decrease in tensile strength. One of the investigations by Swain et al. [54] demonstrated that the tensile strength only decreased when there was weak bonding between fiber and the matrix.

In general, the stiffness property of the SPF/GF reinforced PLA hybrid composites was also determined. Material stiffness property is mainly indicated by tensile modulus. In general, the value of tensile modulus was increased with an increase in wt % of GFs. This statement was valid for both untreated as well as treated SPF/GF reinforced PLA hybrid composite. The tensile moduli for BC treated SPF/GF/PLA hybrid composites were increased by the GF percentage, i.e., for 10, 15, and 20 wt % GF in S9, S8, and S7 composites, the value of tensile moduli were 500, 505, and 607 MPa.

Comparing untreated SPF of S1–S3 composites indicated increasing percentage of GFs improved the tensile modulus of composites. The highest tensile modulus was shown by S2, having 30 wt % of GFs as depicts in Figure 5b. This is since the tensile modulus and strength of GF was higher than SPF. The increment of tensile modulus denoted the improvement of load-bearing capacity.

**Figure 6:**
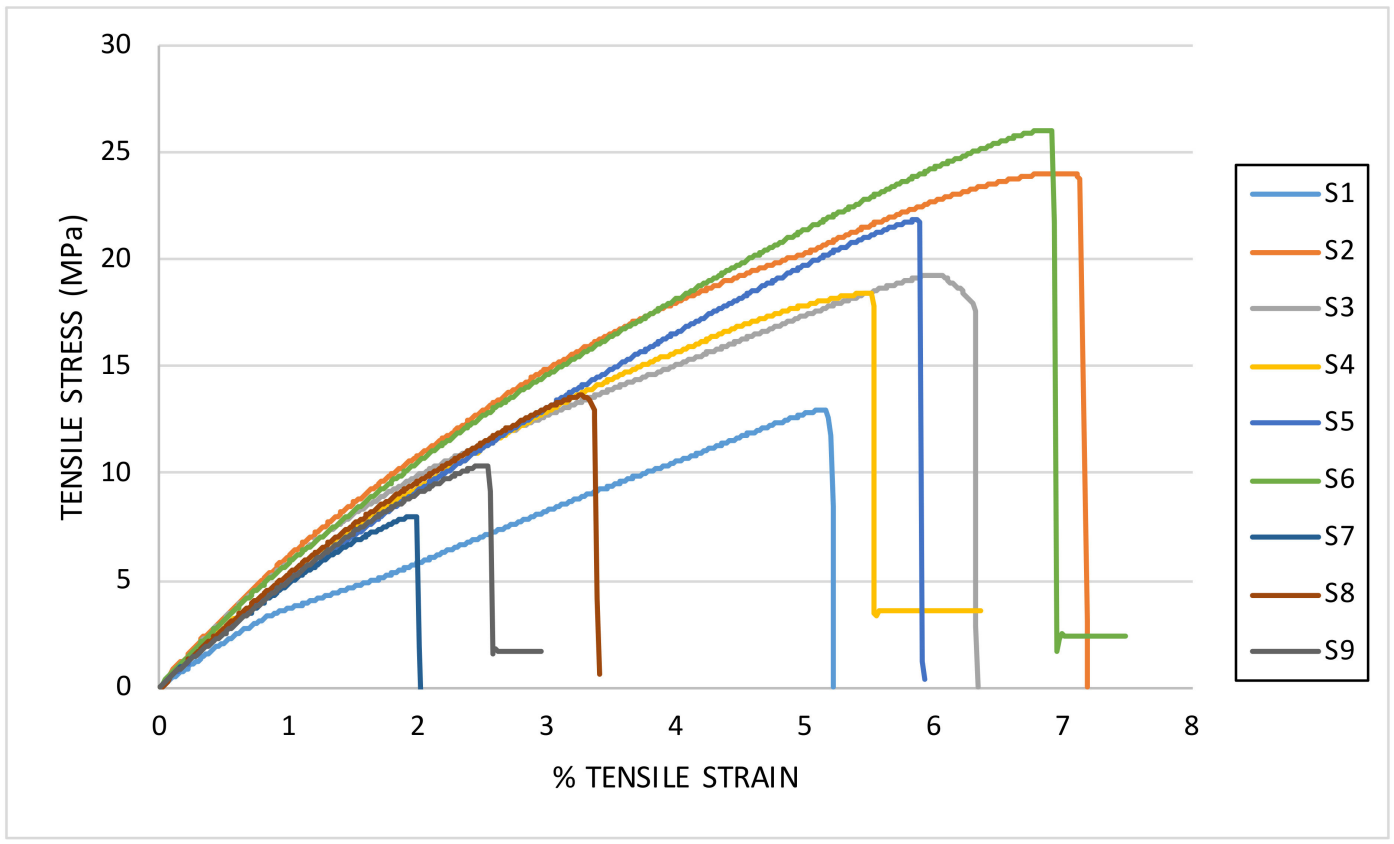
Tensile stress (MPa) versus % tensile strain for various composites.

On comparing the alkaline treated SPF/GF/PLA composites, the analysis showed that the S6 hybrid composite exhibited the maximum tensile modulus of 561 MPa. The S6 hybrid composite had a maximum of 20 wt % of SPF, indicating improvement in the interfacial bonding between SPF and PLA matrix after the alkaline treatment of SPF that consequently resulted in the improved tensile modulus [20]. In addition, after alkaline treatment, rougher topography might cause the qualitative interface between fiber and matrix due to the removal of waxy and impurity substances. A similar increment of tensile modulus after alkaline was revealed by Mukhtar et al. [18] for SPF/GF-reinforced polypropylene composites. Comparing the alkaline, BC treated, and untreated SPF/GF/PLA hybrid composites, it might be noted that BC treatment increased the tensile modulus of the same wt % composite, i.e., S3, S5, and S8. Among these three composites having 15/15/70 wt % (SPF/GF/PLA), the highest value of tensile modulus 505 MPa was shown by S8, followed by 435 MPa and 423 MPa, for S5 and S3 composites, respectively. Previous studies [29,51,55] indicated that the treatment of fibers with BC enhanced the mechanical contact surface with the matrix, which enhanced the interfacial bonding between fiber and matrix and increased stress transfer and tensile modulus of the entire composite. The maximum tensile modulus was shown by the S7 (BC treated) hybrid composite. The tensile properties in terms of the tensile stress–strain curve of various composites is shown in Figure 6.

### 4.6. Flexural Testing

**Figure 7:**
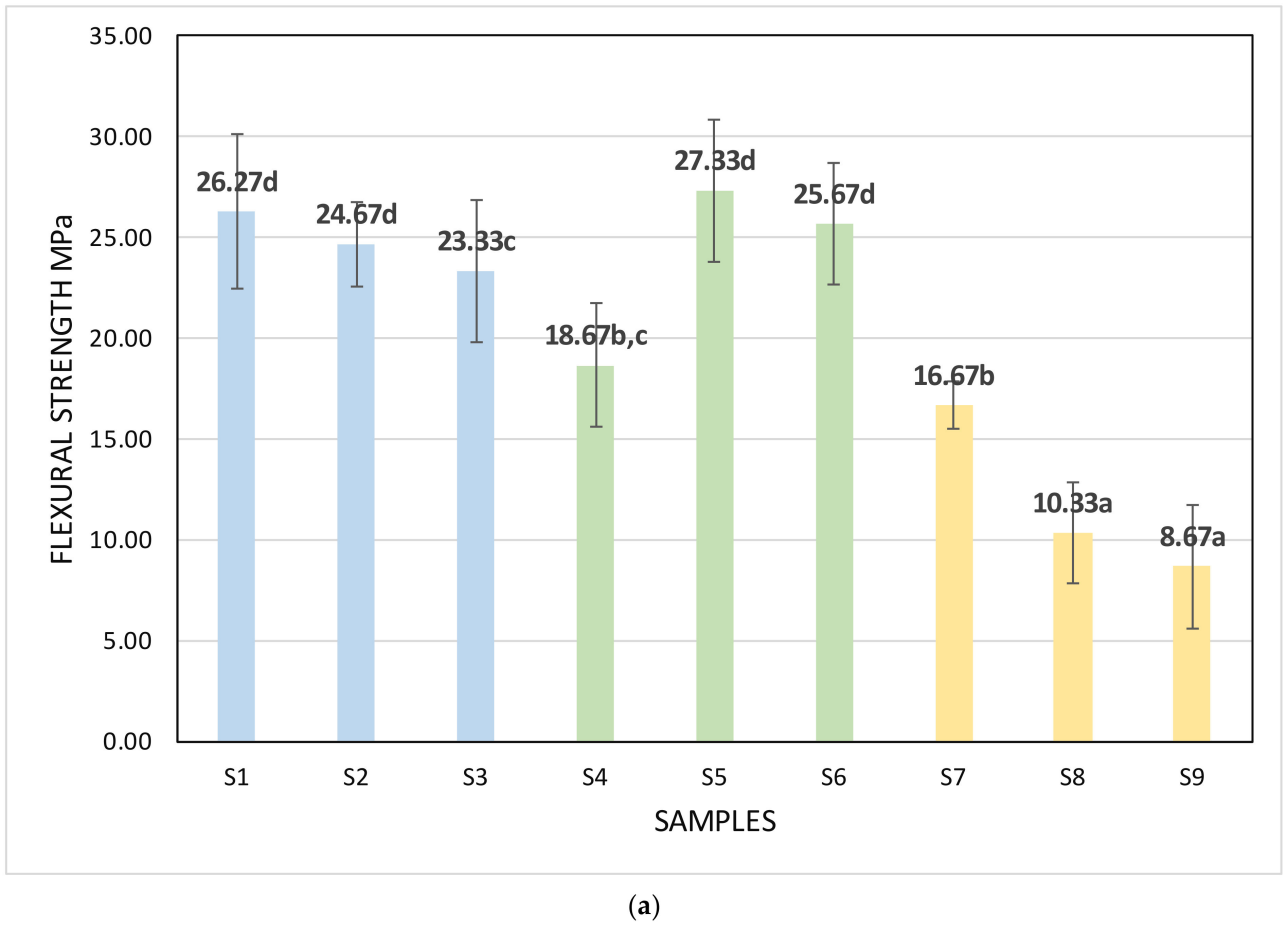

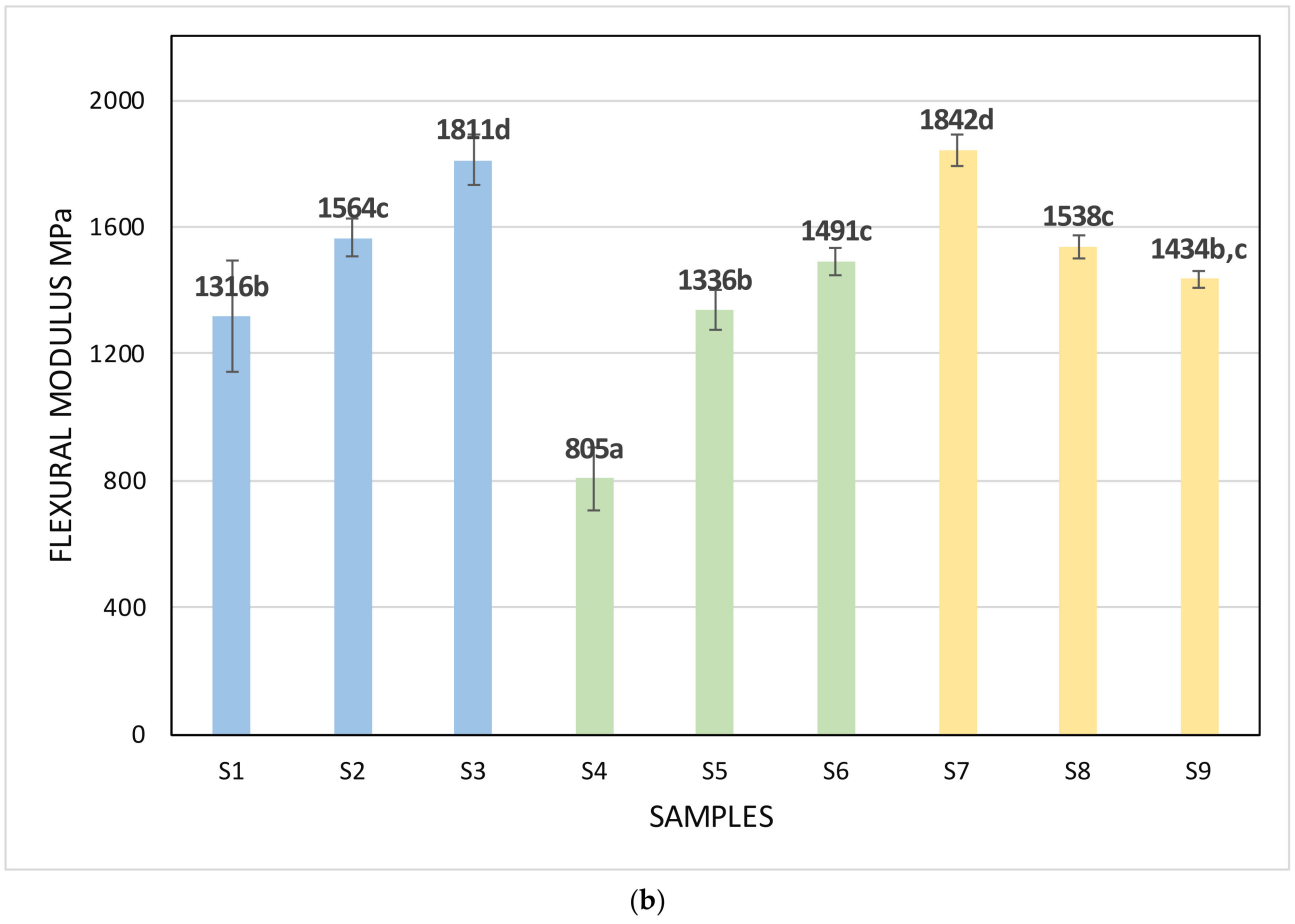
(**a**) Flexural strength versus samples of non-hybrid and hybrid SPF/GF/PLA composite. (**b**) Flexural modulus versus samples of non-hybrid and hybrid SPF/GF/PLA composite. * Values with different letters in the figures are significantly different (*p* < 0.05).

Figure 7a,b shows the effect of treatment on flexural strength and modulus of non-hybrid and hybrid SPF/GF/PLA composites. The bending results analysis confirmed the flexural strength of the composite. In the S5 hybrid composite, the alkaline treatment of SPF enhanced the flexural strength. Comparing S3, S5, and S8 hybrid composite having the same wt % (SPF/GF/PLA-15/15/70), the S5 (alkaline treated) hybrid composite showed maximum flexural strength of 27.3 MPa among all the non-hybrid or hybrid composites. The incorporation of alkaline treated 15 wt % of SPF led to improved flexural strength of the S5 hybrid composite. After the alkaline treatment, about 17% of flexural strength was increased compared with the S3 (untreated) hybrid composite. The increasing of flexural strength after the alkaline treatment was due to partial elimination of hemicellulose, wax, as well as disruption —OH bonding on the fiber, which ensured a better adhesion bonding between the PLA matrix and SPF, whereas the S3 hybrid composite showed low flexural strength caused by poor interfacial bonding between the PLA matrix and SP fiber. This finding was comparable with the SPF/GF-reinforced polypropylene investigated by Mukhtar et al. [18]. Another research highlighted that the fiber treatment plays an important role for flexural strength value. The alkaline treatment of fiber can reduce the cell wall thickening, leading to improved adhesion between fiber and matrix [3]. Similar research reported the effect of alkaline treatment on mechanical properties for roselle/SPF reinforced TPU hybrid composites [20]. Atiqah et al. [3] reported that the flexural strength of SPF hybrid composites also depends on types of surface treatment. The contrast results of alkaline treatment with BC treatment for SPF/GF/PLA hybrid composites are shown. The value of flexural strengths for all three hybrid composites treated with BC were less than the untreated ones.

For non-hybrid S1 composite (only 30 wt % untreated SPF and 70 wt % PLA), the flexural strength was 26.3 MPa, which was supported by the study of Sherwani et al. [40] for flexural analysis of different ratio SPF/PLA composite. The analysis showed that the hybridization of GF did not significantly affect the flexural strength of composites. In the case of S2, it decreased by means of bending ability decrease on the addition of GF. Comparing with untreated SPF/GF, it was clear that the hybridization of the composite resulted in a good flexural modulus of 1811 MPa (S3 hybrid composite), while the flexural moduli for non-hybrid S1 and S2 composite were very low of 1317 MPa and 1564 MPa, respectively. From the comparison of the flexural modulus after alkaline and BC treatment for same wt % hybrid composites (i.e., S5 with S8), it was observed that after BC treatment, the flexural modulus was increased from 1336 MPa to 1536 MPa. BC treatment reduces the diameter of SPF as well as eliminate the lignin and wax layer on fiber. The compatibility between fiber and matrix increased due to the benzene rings’ availability in the benzoyl group attached to the fibers.

Safri et al. [28] also reported the same reason for an increment of flexural modulus after BC treatment of SPF/GF/epoxy hybrid composites. On comparing S6 and S9 having the same wt % ratios (20/10/70) SPF/GF/PLA hybrid composites, almost the same flexural modulus about 1450 MPa was observed in all composites. This value is maximum among alkaline treated SPF, which was proved by Radzi et al. [20] that the alkaline treatment of SPF would improve flexural modulus due to better wetting and good interfacial bonding between matrix and the treated fiber. S7 exhibited 1847 MPa, the highest value of flexural modulus. This was due to various changes at the surface of SPF after BC treated that increased the adhesion between treated fiber and matrix and mechanical interlocking by distributing several small voids on the fiber surface and creating extra fiber interpenetration to the interface. A similar reason was reported for studying the effect of 6% alkaline treatment of SPF for roselle/SPF reinforced TPU hybrid composites [20].

Safri et al. [28] also reported the same reason for an increment of flexural modulus after BC treatment of SPF/GF/epoxy hybrid composites. On comparing S6 and S9 having the same wt.% ratios (20/10/70) SPF/GF/PLA hybrid composites, almost the same flexural modulus about 1450MPa was observed in all composites. This value is maximum among alkaline treated SPF, which was proved by Radzi et al. [20] that the alkaline treatment of SPF would improve flexural modulus because of better wetting and good interfacial bonding between matrix and the treated fibre. S7 exhibited 1847MPa, the highest value of flexural modulus. This was due to various changes at the surface of SPF after BC treated that increased the adhesion between treated fibre and matrix and mechanical interlocking by distributing several small voids on the fibre surface and creating extra fibre interpenetration to the interface. A similar reason was reported for studying the effect of 6% alkaline treatment of SPF for roselle/SPF reinforced TPU hybrid composites [20]. 

### 4.7. Morphological Investigations

**Figure 8:**
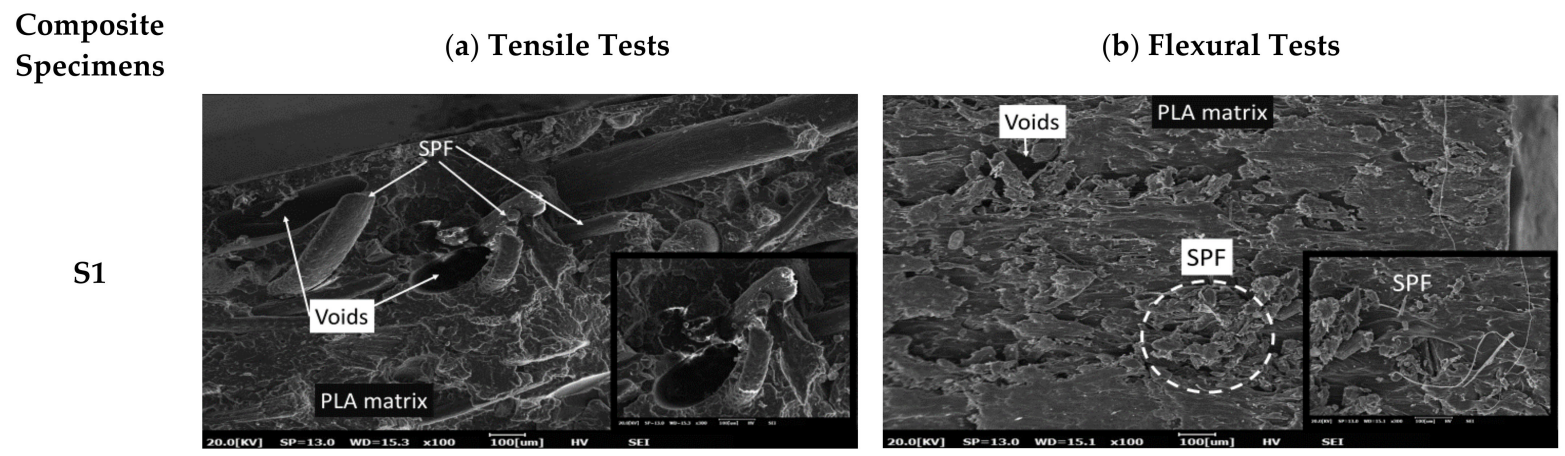

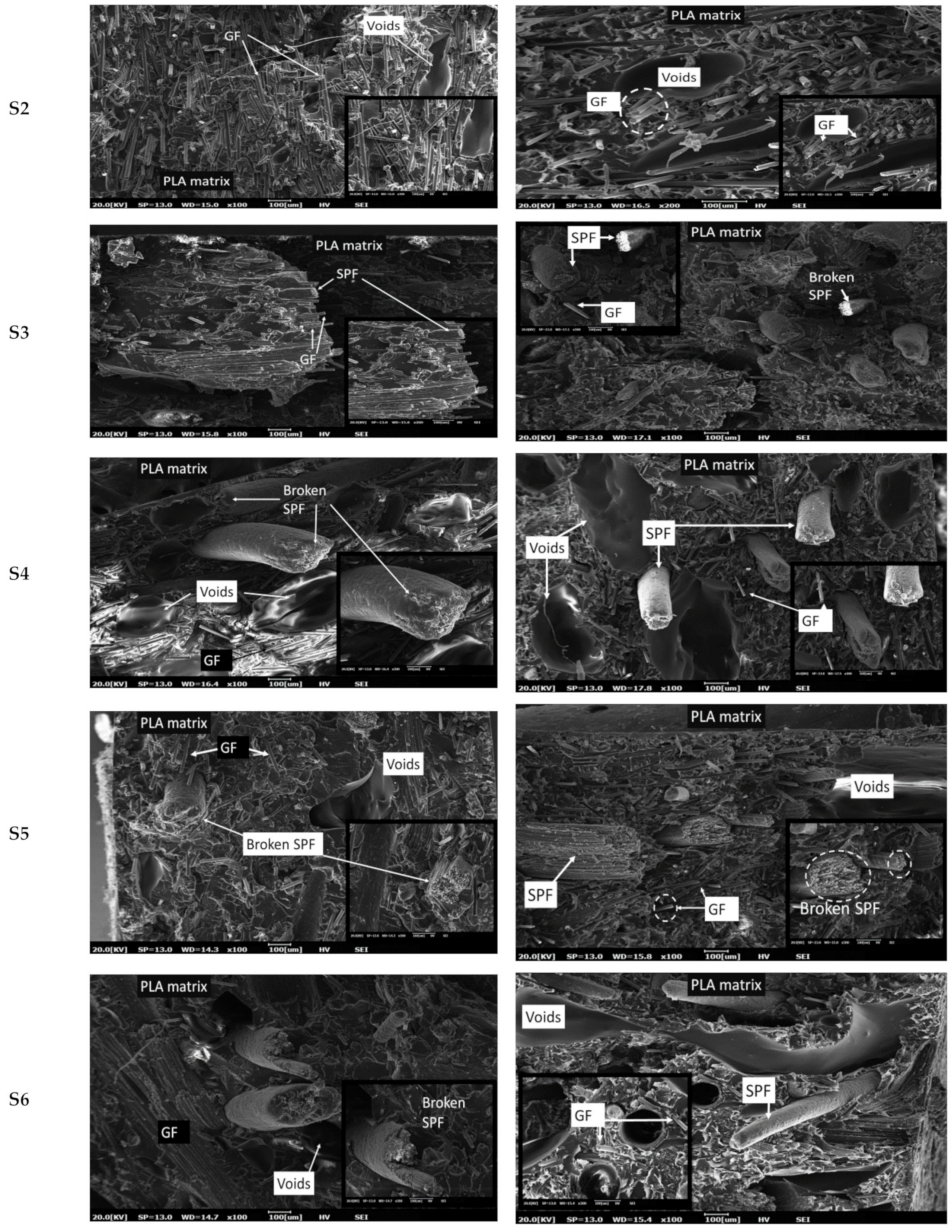

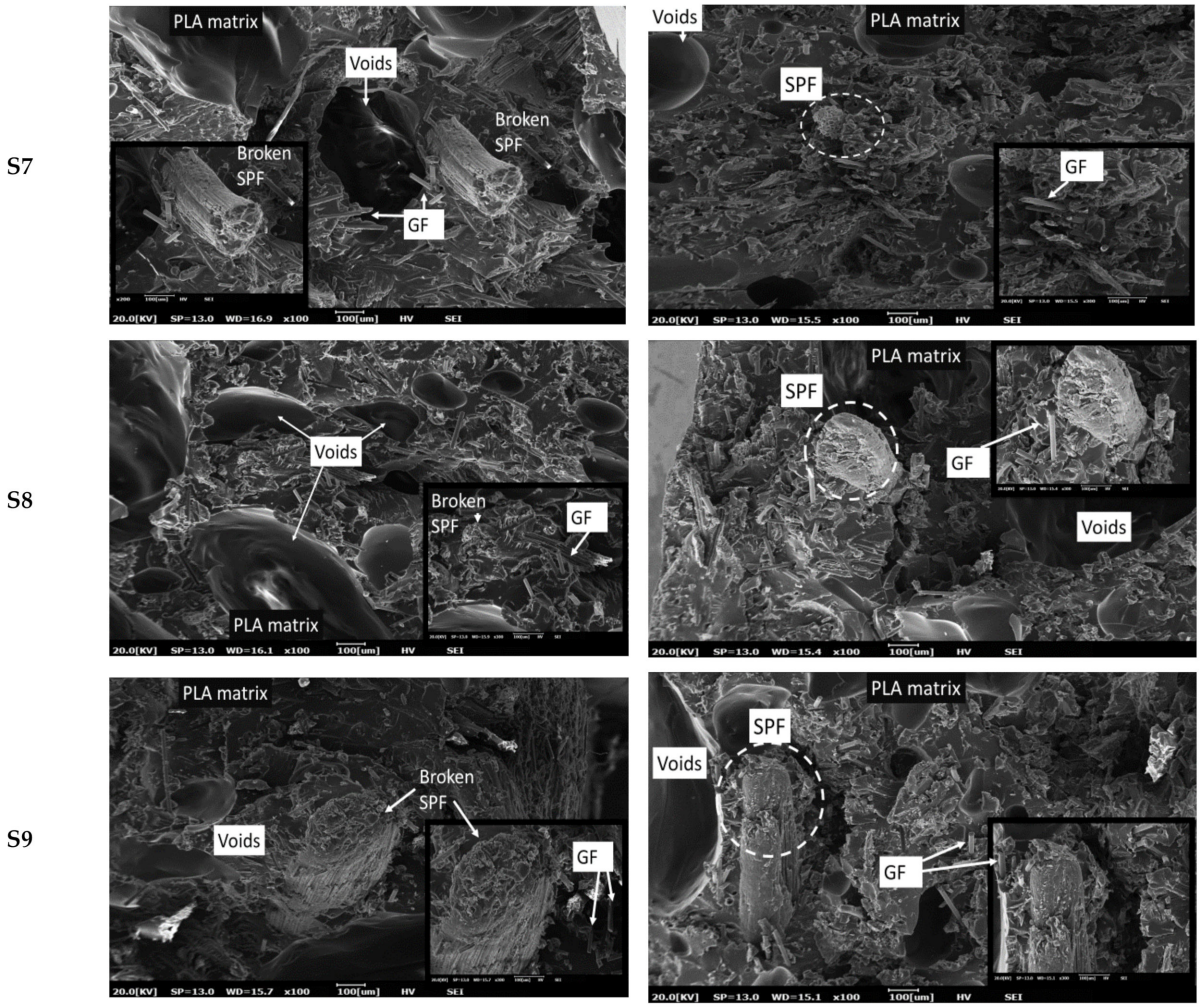
Morphological investigations for fractured surface analysis of (**a**) tensile and (**b**) flexural test. The SEM images clearly defined the presence of voids, breakage of SPF, GF, and PLA matrix.

The morphological analysis was conducted from two different perspectives: tensile and flexural testings of the fractured cross-sectional area, as shown in Figure 8. The analyses were carried out from 100× magnification to 300× magnification for both non-hybrid and hybrid SPF/GF/PLA composites from S1 to S9. In SEM morphology, S1 (untreated/non-hybrid) composites showed that the SPF demonstrated pull-outs, voids, were also visible that indicated poor compatibility with the PLA matrix. Due to the untreated SPF, some wax and lignin contents were visible, and these SEM images were identical to date palm leaf (DPL) images studied by Swain et al. [54], showing the untreated DPL’s lignin and wax contents. Non-hybrid S2 composite revealed that GF was stretched when the tensile load was applied and fewer voids were observed, proving good tensile strength and modulus compared with S1 and S3 composites. Atiqah et al. [36] also mentioned that when the tensile load is applied to SPF/GF/TPU composites, GF stretching occurred. SEM images of the S3 hybrid composite revealed that SPF breakage began when analysing the flexural fracture surface, whereas the other two flexural fracture surfaces of S1 and S2 did not reveal any broken fibres. As a result, the flexural modulus of S3 hybrid composite was the highest among S1, S2, and S3 hybrid composites. SEM figures generally showed a good bonding between alkaline and BC treated fibres and PLA matrix compared with untreated SPF. S4, S5, and S6 (alkaline treated) hybrid composites possessed a rough surface than other hybrid composites that contributed to the enhancement of interfacial bonding between SPF and PLA matrix. The rough surface after alkaline treatment of SPF was because of the removal of hemicellulose, lignin, and waxy layer, where this part was visible on SEM images. Because of this reason, S4, S5, and S6 tensile and flexural fracture surfaces showed broken SPF, which indicated the increments in the tensile and flexural strengths after alkaline treatment. This phenomenon might be due to the rougher surface of SPF, the increased bonding strength between SPF and PLA matrix, and few visible voids on the surface. Breakage of the SPF indicated more energy dissipation when tensile or flexural loads were applied. SPF did not pull out, as proven by the breakage images of SPF in all three alkalines treated S4, S5, and S6 hybrid composites. Similar results were reported by Radzi et al. [20] for alkaline surface-treated roselle fibre/SPF hybrid composite that demonstrated better adhesion between treated fibre and matrix. The tensile and flexural properties of the hybrid composites were also enhanced. S7, S8, and S9 hybrid composites showed the big gaps/voids and low bonding strength between SPF and PLA matrix after BC treatment. SEM images also presented visible fibres break and dislocation, as well as voids/gaps, as aforementioned above. The weak interfacial adhesion caused the pull-outs in SPF. The fractured surface observed for S7, S8, and S9 (BC treated) hybrid composites showed broken/breakage SPF attributed to the increase of both flexural and tensile moduli of the hybrid composites. In previous studies, morphological analysis of BC treated fibre also reported that the wettability of treated fibre with matrix was increased [26,56]. 

SEM images revealed that composites of untreated SPF or BC treated had poor interfacial adhesion, as reported by fiber pull-outs and the presence of holes/voids/gaps. On the other side, alkaline treated S4, S5, and S6 hybrid composites were distinguished by fibers breakage that showed strong adhesion between SPF and PLA matrix. This morphological analysis showed that alkaline and BC treatment was able to modify the SPF surface for good adhesion, as reported in other studies [20,51]

### 4.8. Impact Testing

**Figure 9:**
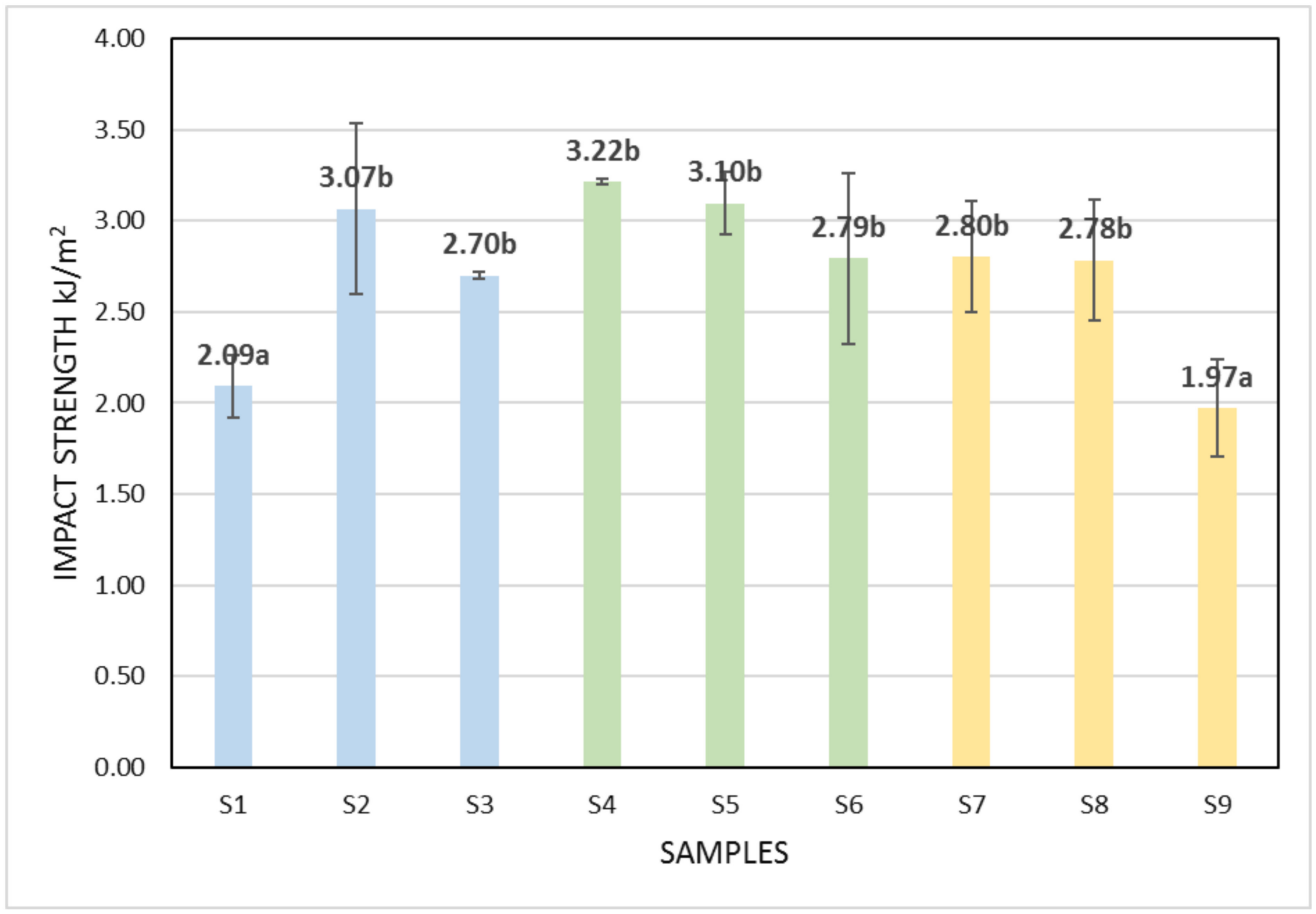
Impact strength versus samples of non-hybrid and hybrid SPF/GF/PLA composite. * Values with different letters in the figures are significantly different (*p* < 0.05).

Table 5 shows tensile strength, tensile modulus, flexural strength, flexural modulus, and impact strength values of non-hybrid and hybrid SPF/GF/PLA composites. Impact strength is used to calculate the dissipation of total energy before ultimate fracture. Figure 9 shows the effect of treatment on the impact strength of non-hybrid and hybrid SPF/GF/PLA composites. Therefore, on hybridization, as wt % of GFs content was increased, the failure mechanism was GF fracture, not GF pull-out, due to the brittle nature of GF. Due to this reason, the composite can withstand a high-speed impact load at higher wt % GFs content. In general, the energy absorption mechanism was not in role, only the energy dissipated in frictional sliding of one fiber with the other due to the interaction of fibers. Moreover, impact strength was increased after hybridization, which increased the stress capabilities. The impact strength of S1 (untreated) composite was 2.09 kJ/m^2^ that increased to 2.70 kJ/m^2^ after the hybridization of GF for S3 hybrid composite. Non-hybrid S2 composite showed good impact strength of 3.07 kJ/m^2^. S4 (alkaline treated SPF) hybrid composite exhibited the maximum impact strength value of 3.22 kJ/m^2^ due to the removals of hemicellulose, lignin, and pectin, wax generation of moisture resistance, and the creation of rough fiber surface after alkaline treatment, which improved the adhesion between treated fiber and matrix. Comparing S4–S6 hybrid composites showed a decrease in impact strength as the wt % of glass content decreased. The impact strength directly depended upon the toughness of the entire composite. Fibers play a crucial role in impact resistance, where the combined effect of both fibers improved their impact strength. Uma Devi et al. [57] reported that as the percentage of GF increased, the impact strength increased for short pineapple fiber/GF/polyester hybrid composites.

Comparing the same wt % hybrid composites, i.e., S3, S5, and S8, S5 (alkaline treated) hybrid composite showed the highest impact value of 3.10 kJ/m^2^ followed by 2.78 kJ/m^2^ for S8 (BC treated) whereas S3 (untreated) exhibited the lowest impact strength value of 2.7 kJ/m^2^. A similar improved impact strength value was reported by Atiqah et al. [3] after alkaline treatment of SPF for SPF/GF/TPU hybrid composites. After S4, the value of impact strength decreased as the SPF content was increased. With an increase in SPF content, the interspaces and stress concentration shoot up, which acted as crack propagation. The same trend of decreasing impact strength value with increasing fiber content was reported by Swain et al. [53] for date palm leaf/GF reinforced hybrid composite.

BC treated SPF also improved the impact strength from 2.70 kJ/m^2^ for S3 (untreated) hybrid composite to 2.80 kJ/m^2^ for S7 (BC treated) hybrid composite. This might be due to good interlocking between treated fiber and matrix, which allowed maximum energy absorption and stopped the crack propagation, enhancing the impact properties [58,59]. Thiruchitrambalam et al. [27] reported a 12% impact strength increment after BC treatment for palmyra palm leaf stalk fiber-polyester composites. Swain et al. [60] also revealed that after BC treatment of jute fiber, the impact strength increased for developing jute/epoxy composites. The lowest value of impact strength of 1.97 kJ/m^2^ was shown by the S9 hybrid composite that might be due to the insufficient resistance to pull out fiber during impact fracture. Fracture of a matrix, fiber/matrix debonding, and fiber pull-out are three main causes for impact failure [60].

### 4.9. Fourier Transform Infrared (FTIR)

**Figure 10:**
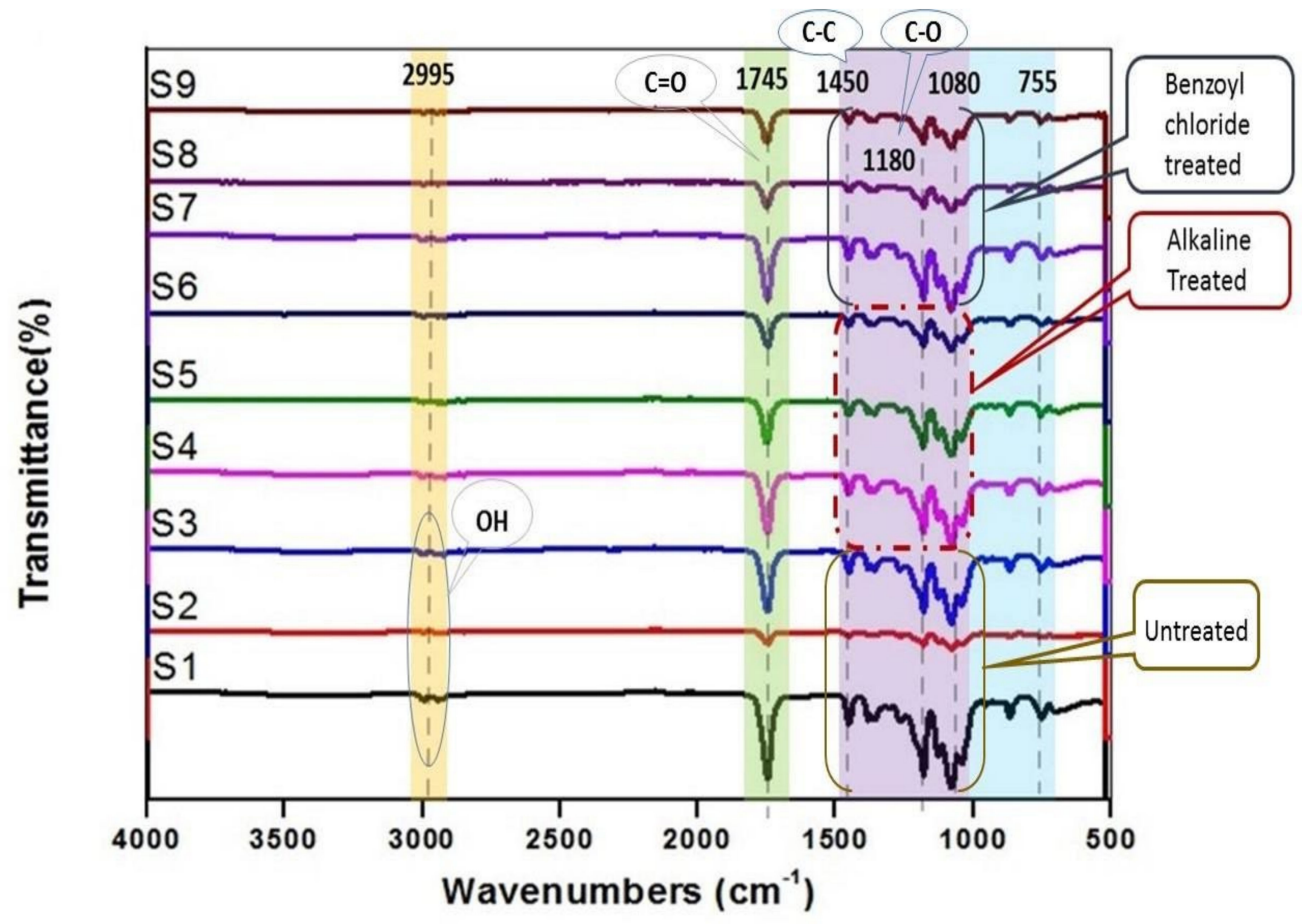
FTIR spectrum detailed data analysis of non-hybrid and hybrid SPF/GF/PLA composites.

Fourier transform infrared (FTIR) spectroscopy is often used to verify the correct mixture of matrix-fiber ratio due to the interplay between components in polymer composites is complex [61,62,63,64,65,66,67,68]. Figure 10 shows the FTIR analysis for untreated, alkaline, BC treated of non-hybrid, and hybrid SPF/GF/PLA composites. From the figure, it is clear that all hybrid composite showed almost similar patterns. This analysis was used to determine the effect of alkaline and BC treatment on SPF and the chemical bonding nature between SPF, GF, and PLA. FTIR of untreated and treated SPF/GF/PLA composites revealed changes in the associated functional groups. Spectrum helps us to determine the presence of lignin, cellulose, and hemicellulose in (C-H rocking vibrations), 1180 cm^−1^ cellulose (C-O-C asymmetric valence vibration, 1316 cm^−1^ cellulose (C-H_2_ rocking vibration), 1370 cm^−1^ cellulose (C-H_2_ deformation vibration), 1424 cm^−1^ cellulose, 1227 cm^−1^ lignin (C-C plus C-O plus C=O stretch) [69,70].

In the range, 1300 cm^−1^ to 1160 cm^−1^ belonged to the C-C group (lignin in-ring stretch mode). The difference in peak heights of the untreated (S1, S3) and alkaline treated (S4–S6) hybrid composites was observed that resulted from the reduced amount of lignin and hemicelluloses in SPF after alkaline treatment. Significant changes at peaks 1180 cm^−1^ were observed, showing C-O in alkaline treated fiber concerning primary alcohol stretching, peak reduction compared to untreated fiber. Other studies have also confirmed this related disappearance of lignin and hemicelluloses after alkaline treatment that improved the adhesion between the fiber and the matrix [3,19,71]. The sharp peak at 1745 cm^−1^ for S1 composite was observed that was associated with the presence of hemicellulose C=O stretching vibration [71]. The intensity changes at the 1756 cm^−1^ peak showed the esterification among the —OH groups of SP fiber and -COOH terminal groups of PLA. A small peak in all nine samples at 2995 cm^−1^ was ascribed with the frequency of the O-H group [72,73]. This intensity was also decreased after treatment of SPF. After 6 wt % alkaline treatment of SPF, the —OH groups were substituted with –ONa groups. According to Bachtiar [71], at 4% alkaline treatment, the nature of SPF changes to hydrophilic since the cellulose I changed to Cellulose II.

The peak at 1450 cm^−1^ revealed C-C stretching in the aromatic ring, and the peak at 1719 cm^−1^ indicated the C=O stretching of the benzoyl carbonyl group in the benzoylated fiber. As the benzoyl group reacted with the —OH group of SPF, the hydrophilic character decreased by reducing the hydroxyl group, which was indicated at 2995 cm^−1^. A similar FTIR result was also reported by Salisu et al. [58] after benzoylation of sisal fiber unsaturated polyester-reinforced composites.

## 5. Conclusions

The physical, mechanical, and morphological properties of treated SPF/GF reinforced PLA hybrid composites were investigated. This novel SPF/GF reinforced PLA hybrid composites exhibited lower densities after alkaline treatment of SPF, improved water absorption, and thickness swelling after both SPF treatments. It was observed that the incorporation of alkaline treated SPF/GF reinforced PLA matrix increased the tensile and flexural strengths. Both alkaline treated S6 and S5 hybrid composites exhibited the highest tensile strength of 26.3 MPa and flexural strength of 27.3 MPa. It was also found that BC treated SPF/GF reinforced PLA hybrid composites exhibited the highest tensile and flexural moduli. S7 hybrid composite recorded the highest tensile and flexural moduli of 607 MPa and 1847 MPa, respectively. Nevertheless, the incorporation of alkaline treated S4 hybrid composite showed the highest value of impact strength of 3.22 kJ/m^2^. This value was reduced as the SPF content was increased. The morphological investigation revealed that alkaline treatment of SPF possessed better interfacial adhesion between SPF and PLA matrix. FTIR results also showed that after alkaline treatment, the adhesion between fiber and matrix was improved. This combination of alkaline and BC treated SPF/GF reinforced PLA hybrid composite resulted in good physical and mechanical properties. Therefore, this composite can be proposed for the fabrication of automotive components. Future research will primarily focus on replacing Acrylonitrile butadiene styrene (ABS) plastic motorcycle battery housing parts with these hybrid composites.

## Figures and Tables

**Table 1:** Some typical properties of E-glass fiber [42].

Liquidus Temp. (°C)	1140
Fiberising temp. (°C)	1200
Tensile strength at 25 °C (GPa)	3.7
Tensile modulus (GPa)	76
Density (g/cm^3^)	2.53
Refractive index	1.550
Volume resistivity (Ωcm)	10^15^
Dielectric constant at 25 °C and 10^6^ Hz	6.6
Loss tangent at 25 °C and 10^10^ Hz (10^−3^)	3.9

**Table 2:** Composition of alkaline treated SPF.

	Cellulose (%)	Hemi-Cellulose (%)	Holo-Cellulose (%)	Lignin (%)	Extractive (%)	Ash (%)	References
SPF	43.88	7.24	51.12	33.24	2.73	1.01	[9]
Alkaline treated SPF (5% *w*/*v* NaOH solution)	82.33	3.97	86.3	0.06	-	0.72	[43]

**Table 3:** Formulation of non-hybrid and hybrid composites.

No. of Samples	Matrix	Reinforcement		
	PLA (wt %)	SPF		GF (wt %)
		Treatment	(wt %)	
S1	70	-	30	0
S2	70	-	0	30
S3	70	-	15	15
S4	70	6% NaOH	10	20
S5	70	6% NaOH	15	15
S6	70	6% NaOH	20	10
S7	70	15 min BC	10	20
S8	70	15 min BC	15	15
S9	70	15 min BC	20	10

BC—Benzoyl Chloride.

**Table 4:** Moisture contents, densities, and void contents of non-hybrid and hybrid SPF/GF/PLA composites.

Composite Specimens	Moisture Contents	$\rho_{ct}$ (gr/cm^3^)	$\rho_{comp.\;\;exp.}\;(\mathrm{gr}/{\mathrm{cm}}^{3})$	Void Contents $V_{void}$
S1	1.79%	1.23	1.21 ± 0.039 ^a,b^	1.94%
S2	0.75%	1.40	1.29 ± 0.066 ^b,c^	7.9%
S3	0%	1.34	1.32 ± 0.029 ^c^	1.63%
S4	1.24%	1.38	1.32 ± 0.045 ^c^	3.93%
S5	2.09%	1.34	1.31 ± 0.011 ^b,c^	2.45%
S6	1.07%	1.30	1.16 ± 0.062 ^a^	1.5%
S7	1.29%	1.38	1.30 ± 0.072 ^b,c^	7.5%
S8	0%	1.34	1.19 ± 0.062 ^a^	9.7%
S9	0%	1.30	1.22 ± 0.044 ^a,b^	6.5%

* Values with different letters in the same column are significantly different (*p* < 0.05).

**Table 5:** Tensile, flexural, and impact properties of non-hybrid and hybrid SPF/GF/PLA composites.

Composite Specimens	Tensile Strength (MPa)	Tensile Modulus (MPa)	Flexural Strength (MPa)	Flexural Modulus (MPa)	Impact Strength (kJ/m^2^)	References
SP30	13.70	-	26.65	-	1.99	[40]
SA	17.83 ± 2.43	431 ± 43	32.34 ± 2.92	1209 ± 258	4.39	[41]
SB	7.01 ± 0.8	602 ± 7	6.07 ± 0.83	863 ± 106	2.39	[41]
S1	16.0 ± 2.64 ^b,c^	433 ± 30 ^a^	26.3 ± 3.85 ^d^	1316 ± 175 ^b^	2.09 ± 0.15 ^a^	Current study
S2	23.7 ± 3.21 ^d,e^	539 ± 14 ^b,c^	24.7 ± 2.08 ^d^	1564 ± 60 ^c^	3.07 ± 0.2 ^b^	Current study
S3	11.7 ± 3.51 ^a,b^	423 ± 16 ^a^	23.3 ± 3.51 ^c^	1811 ± 80 ^d^	2.70 ± 0.10 ^b^	Current study
S4	18.7 ± 4.93 ^c,d^	517 ± 7 ^b,c^	18.7 ± 3.05 ^b,c^	805 ± 100 ^a^	3.22 ± 0.10 ^b^	Current study
S5	14.0 ± 4.00 ^a,b,c^	435 ± 15 ^a^	27.3 ± 3.52 ^d^	1336 ± 64 ^b^	3.10 ± 0.14 ^b^	Current study
S6	26.3 ± 2.51 ^e^	561 ± 16 ^c,d^	25.7 ± 3.05 ^d^	1491 ± 45 ^c^	2.79 ± 0.39 ^b^	Current study
S7	11.0 ± 3.00 ^a,b^	607 ± 60 ^d^	16.7 ± 1.15 ^b^	1842 ± 50 ^d^	2.80 ± 0.25 ^b^	Current study
S8	9.3 ± 1.52 ^a^	505 ± 25 ^b^	10.3 ± 2.51 ^a^	1538 ± 35 ^c^	2.78 ± 0.27 ^b^	Current study
S9	10.3 ± 3.05 ^b^	500 ± 20 ^b^	8.7 ± 3.05 ^a^	1434 ± 29 ^b,c^	1.97 ± 0.22 ^a^	Current study

* Values with different letters in the same column are significantly different (*p* < 0.05). SP30 defined for Untreated SPF (30%)/PLA (70%). SA defined for 6% conc. of NaOH treated SPF (30%)/PLA (70%). SB defined for 15 min soaking BC treated SPF (30%)/PLA (70%).

## Data Availability

The data that support the findings of this study are available from the corresponding author, upon reasonable request.
